# Understanding Aging Mechanism of SBS/CR Composite Modified Asphalt Based on ATR-FTIR: Chemical Degradation and Aging Deterioration

**DOI:** 10.3390/ma19010167

**Published:** 2026-01-02

**Authors:** Lin Li, Chen Yang, Lingwen Li, Weiwen Quan, Yuanxiang Wang, Yiqiu Tan, Yunliang Li, Zhenyu Zhang

**Affiliations:** 1 School of Transportation Science and Engineering, Harbin Institute of Technology, Harbin 150090, China; 13810455287@163.com (L.L.); lilingwen0912@163.com (L.L.); liyunliang@hit.edu.cn (Y.L.); 2 Center of Engineering Technology and Materials Research, China Academy of Transportation Sciences, Beijing 100013, China; 3 School of Civil Engineering, Central South University of Forestry and Technology, Changsha 410004, China; quanweiwen@csuft.edu.cn; 4 Xinjiang Transportation Investment Duku Expressway Investment and Development Co., Ltd., Urumqi 830009, China; omitsmilem@163.com; 5 CATS Testing Technology (Beijing) Co., Ltd., Beijing 101300, China; zzy16050@163.com

**Keywords:** polymer modifiers, composite modified asphalt, infrared spectroscopy, chemical reactions, aging mechanism

## Abstract

**Highlights:**

**What are the main findings?**

- SBS and CR modifiers exhibit significant yellowing degradation after aging.
- Some chemical bonds in asphalt are easily broken to produce reactive free radicals under aging.
- Free radicals can react chemically to produce sulfoxide and carbonyl groups.
- The aging reaction of the asphalt is a dual sequential oxidation process.

**What are the implications of the main findings?**

- Exploring the aging chemical reaction pathway of polymer-modified asphalt.
- Providing targeted guidance for anti-aging modified asphalt.

**Abstract:**

To explore the aging mechanism of (Styrene Butadiene Styrene) and CR (Crumb Rubber) composite-modified asphalt in a multi-source environment, the characteristics of functional group changes in the infrared spectroscopy of SBS and CR modifiers as well as their single and composite modified asphalts under thermal, UV, and coupled aging were tested using Attenuated Total Reflection–Fourier Transform Infrared Spectroscopy (ATR-FTIR) technology. It was found that SBS and CR modifiers exhibited significant yellowing degradation after aging due to high-energy effects, causing abstraction of α-hydrogen from polybutadiene via oxidation, initiating radical chain reactions. The addition of SBS and CR to asphalt significantly increased the absorption peaks of 966 cm^−1^ polybutadiene and 699 cm^−1^ polystyrene. However, certain labile bonds in the modified asphalt, such as the C-H bond, C-C bond, and C=C double bond in polycyclic aromatic hydrocarbons, were easily broken to produce reactive free radicals under aging, which reacted chemically with other components to produce new sulfoxide and carbonyl groups. Overall, the aging reaction of the asphalt was a dual sequential oxidation process. Under normal temperature conditions in the early stage, a large number of sulfoxides were oxidized. In the later stage of the reaction, as the concentration and persistence of active free radicals increased, the oxidation reaction of the asphalt benzyl carbon also enhanced significantly, ultimately generating carbonyls.

## 1. Introduction

As typical and widely used polymer modifiers, the addition of Styrene-Butadiene-Styrene block copolymer (SBS) and Crumb Rubber (CR) can form new stable and elastic colloidal structures in asphalt. In particular, the combination of these two modifiers can significantly improve the overall road performance of asphalt and compensate for any defect in the individual modifiers if the asphalt is modified separately, thus improving the stability and durability of the composite modified asphalt system [1,2]. However, due to the unsaturated C=C bonds of the modifier molecules at the microscopic chemical composition level, α-hydrogen atoms become highly susceptible to oxidation under thermal or UV stress, and the anti-aging performance of the modified asphalt in multi-source environments is not favorable [3]. Exploration of the aging mechanism of modified asphalt is of great significance for further upgrading its long-term service performance.

Fourier Transform Infrared Spectroscopy (FTIR) is an important method for analyzing the characteristics of the functional groups of organic compounds. It identifies the molecular characteristic spectra of different chemical structures in asphalt binders by detecting transmission or reflection signals [4,5]. The infrared absorption peak has a very strict correspondence with the chemical structure of substances. Various functional groups have a great influence on the position and intensity of the infrared absorption peak and exhibit different characteristics on the infrared spectrum. The FTIR method can be used for fundamental research on molecular structures, and for qualitative and quantitative analysis of the chemical composition of substances [6]. Compared to traditional testing methods, FTIR offers several advantages, including convenience, speed, non-destructive analysis, and no pollution. These benefits make FTIR a valuable tool for evaluating the structure and performance of asphalt [7]. The addition of polymer modifiers, such as SBS and CR, adds some new characteristic absorption peaks to the infrared spectrum of asphalt [8,9]. Under the influence of environmental conditions, such as thermal oxygen and ultraviolet radiation, significant changes may also take place in the spectrum, especially in the absorption peak of the fingerprint area (1300~400 cm^−1^), which would help in distinguishing small differences in asphalt under different conditions. It is an effective means to explain the mechanism of asphalt modification and aging. Among them, the Attenuated Total Reflection–Fourier Transform Infrared Spectroscopy (ATR-FTIR) method involves a simple and fast sampling operation, and has obvious advantages for testing asphalt materials, which can be applied in the composition analysis of many modified asphalt materials.

On one hand, ATR-FTIR can quickly evaluate and predict the performance of asphalt and identify its oil sources by significantly reducing the time required to test various indicators of asphalt materials [10,11,12,13,14]. If the positions of the characteristic absorption peaks of the SBS-modified asphalts are the same, but their heights are significantly different, then the content of SBS in any modified asphalt can be detected easily. On the other hand, ATR-FTIR is also a typical method used to reveal whether any chemical change occurred during the modification mechanism of asphalt. By analyzing the infrared spectra of raw asphalt, modified asphalt, and the modifiers, it is possible to determine whether the modification process is primarily driven by a physical change or chemical modification [15,16]. Infrared spectroscopy can be used to analyze the polymer content in modified asphalt and changes in its molecular structure, suggesting that the changes in the performance of modified asphalt are mainly caused by phase changes in the modifier of the asphalt. Additionally, ATR-FTIR is one of the primary methods used to reveal the aging mechanisms of asphalt. For example, it can be used to study the increments in the indices of various functional groups before and after aging in the same asphalt. The increment in the values of the indices of these functional groups represents the degree of asphalt aging [17,18].

SBS/CR composite-modified asphalt is a multiphase composite material; with CR mainly composed of natural rubber, synthetic rubber, carbon black, and additives [19,20]; while asphalt is a complex black-brown mixture composed of hydrocarbons of varying molecular weights and their non-metallic derivatives. Compared to a base asphalt, the aging of SBS/CR composite-modified asphalt includes both the asphalt phase and the modifier phase, making it more challenging to reveal the aging mechanism, due to its complex material composition. For CR, the aging process involves degradation, cross-linking, and compositional changes in the rubber particles. Cross-linking predominates in the initial stage, while degradation becomes more significant in the later stages [21]. During degradation, the NR content decreases most significantly, followed by the synthetic rubber and filler [22]. The degradation mechanism of CR involves the cleavage and formation of C-S and S-S bonds, the former being sensitive to low temperatures, whereas the latter primarily occur in high-temperature environments. With an increasing degree of CR degradation, the thermal stability, low-temperature performance, and processability of the modified asphalt are improved [23]. In case of asphalt, it ages synchronously with CR. The carbonyl and sulfoxide indices obtained through ATR-FTIR are commonly used to indicate the aging degree of the asphalt binder [24], which is observed not only during thermal aging but also during UV aging [25]. Asphalt aging is characterized by increased indices of carbonyl and polybutadiene functional groups, accompanied by a loss of saturates, carbonation, and the degradation of poly-aromatic cores of asphaltenes and resins [26]. Additionally, monitoring of the aliphatic and aromatic indices is also an effective way to characterize the aging degree of asphalt [27,28]. As the aging process continues, CR continuously degrades, resulting in a reduction in the light fraction absorbed by the rubber, leading to an increase in the asphalt phase. The degradation of CR further enhances the interaction between the rubber and asphalt. The difference in the material functions leads to dynamic changes in the thermo-oxidative aging reaction [29,30]. For the SBS-modified asphalt, the polybutadiene index gradually decreases, while the carbonyl index increases, which is a commonly recognized aging phenomenon [31,32,33]. It is believed that SBS primarily undergoes degradation during aging, mainly because of C=C bonds and *α*-H in its triblock copolymer structure, which are prone to oxidative chain scission, forming a diblock copolymer structure.

However, the complexity of the simultaneous aging of the asphalt phase and the polymer in SBS/CR composite-modified asphalt limits our understanding of its aging mechanism. During the aging process of the SBS/CR composite-modified asphalt, the aging of the asphalt, SBS, and CR occurs simultaneously. The aromatic components and resin in the asphalt undergo condensation reactions to form secondary resin and secondary asphaltene, which gradually harden. The SBS and CR modifiers undergo softening due to desulfurization and degradation. The interaction between these hardening and softening processes leads to unclear aging mechanisms in the SBS/CR composite-modified asphalt. On this basis, in this paper, aging is applied to modifiers, single-modified asphalt, and composite-modified asphalt simultaneously, and then the ATR-FTIR method is applied to quickly and efficiently analyze the functional groups of the materials before and after aging. Finally, the aging mechanisms of modifiers and modified asphalt in composite modified asphalt are revealed from the perspectives of aging oxidation and chemical degradation. These findings would provide a theoretical basis for tracking the aging path of each component of SBS/CR composite-modified asphalt and have important reference significance for proposing targeted anti-aging measures for composite-modified asphalt.

## 2. Materials and Methodologies

### 2.1. Subsection

The raw materials used in this research mainly included base asphalt, SBS, CR, plasticizer, and stabilizer. Among them, the base asphalt was collected from Liaohe Petrochemical 90# asphalt (Panjin, China). SBS was YH-791 (SBS 1301). CR was a desulfurized rubber powder prepared through thermal–mechanical extrusion and shear processes, which helped to improve the storage stability of the modified asphalt. Plasticizer was an organic substance that supplemented the light component of the asphalt. The stabilizer was mainly composed of sulfur, which was a reactive stabilizer. The basic properties of each raw material are listed in Table 1.

### 2.2. Preparation Process of Composite Modified Asphalt

**Figure 1:**
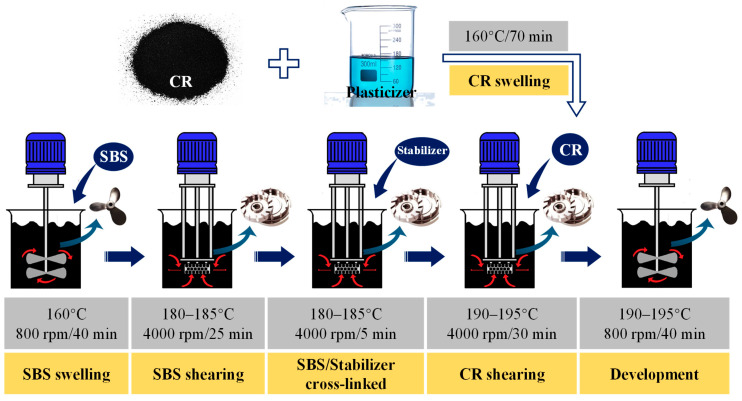
Preparation process of modified asphalt.

In this study, the composite modified asphalt was prepared as shown in Figure 1, which mainly included five processes: (1) SBS swelling, (2) SBS shearing, (3) CR swelling, (4) CR shearing, and (5) development of the final composite-modified asphalt. In addition, the following three materials were also prepared: SBS-modified asphalt, CR-modified asphalt, and SBS/CR composite-modified asphalt. All these modified asphalt materials were prepared as above by removing the corresponding material mixing link according to the principle of equal amount splitting. The proportions of these modified asphalt materials are listed in Table 2.

### 2.3. Aging Methods for Asphalt Materials

Based on previous research, the aging of all five asphalt materials was tested by simulating their aging behaviors under actual service conditions, which mainly included the following three processes:

(1) Thermal oxygen aging (simulates the aging behavior of asphalt during heating, storage, and transportation): Low-speed heating and stirring for 6 h at a temperature of 90 °C above the material softening point, with a stirring rate of 800 rpm.
(2) UV aging (simulates the aging behavior of asphalt pavement under long-term service conditions): Irradiation in a UV aging chamber with an intensity of 130 W/m^2^ for 220 h and a temperature of 40 °C. The ultraviolet radiation type in this study is UVA-365, with a wavelength range of 320–400 nm.
(3) Coupled aging (the effect of water): First thermal oxygen aging, then UV aging, and finally, immersion in water for 10 days.

### 2.4. Testing Method of ATR-FTIR

In this study, the Thermo Nicolet iS50 Fourier infrared spectrometer (manufacturer: ThermoFisher company from Waltham, MA, United States) and ATR mode were used, with a wavelength ranging from 4000 cm^−1^ to 400 cm^−1^ in the mid infrared region, for effectively describing the chemical composition of polymer molecules in the modified asphalt, further explaining the aging mechanism of the SBS/CR composite-modified asphalt.

## 3. Aging Mechanism of Polymer Modifiers

In addition to the SBS/CR composite-modified asphalt and other asphaltic materials, SBS and CR polymer modifiers were passed through the same thermal, UV, and coupled aging processes to observe the changes in both their macro and micro states and the differences in compositions of the polymer modifiers when subjected to aging alone. The two materials were laid flat on the bottom of an aluminum box and aged in thermal, UV, and coupling environments in that order.

### 3.1. Macroscopic Physical States

**Figure 2:**
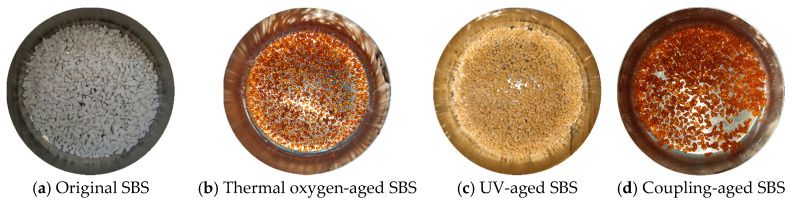
Macro state pictures of SBS under different aging conditions.

**Figure 3:**
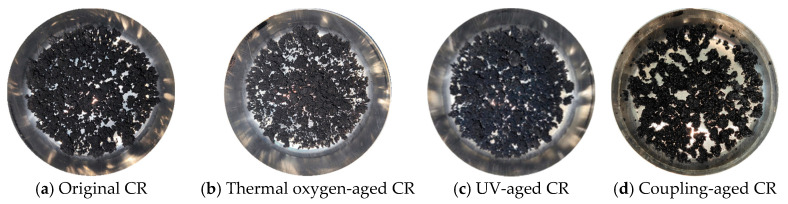
Macro state pictures of CR under different aging conditions.

The results of the macroscopic state of the SBS and CR polymer modifiers obtained after different aging tests are shown in Figure 2 and Figure 3, respectively. From Figure 2a to Figure 2d, it can be seen that the original SBS modifier was white, with thermoplastic elastomer particles. After high-temperature heating and aging, the SBS particles became orange-yellow, transparent, hard, reduced in volume, and bonded into blocks, which adhered to the bottom of the sample box. After UV aging, the SBS particles turned yellow with a brittle texture without elastic characteristics, making them easy to grind into powder. However, they still had a loose particle structure without the phenomenon of bonding into blocks. The state of SBS after the coupling aging was similar to that after the thermal oxygen aging, indicating that even after undergoing UV radiation and water aging successively, the SBS was dehydrated and aged into a relatively hard bonding state after the thermal oxygen aging, and its macroscopic state was stabilized. Therefore, the effects of UV and water were not significant at this time. Figure 3a–d showed that the original CR was a black elastomer. After high-temperature heating and aging, its blackness decreased, and it became too hard to deform. Since the original CR was black, there was no significant change in its macroscopic state after the UV radiation and water aging. After water aging, CR still remained in a similar state to that after the thermal oxygen aging, with a hard and brittle texture.

The phenomenon of turning yellow and transparent after aging, such as that seen in SBS, is commonly referred to as the yellowing of polymers. The main components of SBS are styrene-butadiene-styrene ternary block copolymers, which belong to the class of aromatic polymers. These polymers are very sensitive to high-temperature thermal effects and UV radiation and are prone to yellowing. The reason is that these external environments degrade the polymers, usually in the following three steps:

(1) Initiation: The energy generated during the heat treatment and ultraviolet irradiation directly leads to the degradation of polymers, producing free radicals and increasing the kinetic energy of the degraded polymers. These free radicals react and form stable degradation compounds, which sometimes exhibit adverse characteristics such as changes in odor or color (especially yellowing), as well as phenomena such as embrittlement, loss of mechanical properties, and generation of new free radicals.
(2) Autocatalytic continuous degradation: Small polymer fragments containing new free radicals are abnormally active. As the concentration of these fragments increases during the initiation stage, the reaction becomes self-sustaining until it enters a continuous degradation state of linear autocatalysis. At the same time, some independent reactions also take place in the automatic catalytic cycle, such as free radical recombination, cross-linking, side branching, primary and secondary polymerization chain breaking, secondary or tertiary hydrogen atom abstraction, oxidation and radical formation, C=C unsaturation, and formation of a polyene sequence.
(3) Ending: Finally, due to the depletion of new free radicals or the exceeding of the service life of the polymers, such as SBS, this adverse chemical reaction of degrading the polymers is reduced.

Accordingly, stabilizers are usually added during the preparation of polymer-modified asphalt, such as asphalt modified with SBS and CR. The role of the stabilizers is not only to prevent the segregation of the polymers in the asphalt but also to prevent their degradation to some extent. Most polymer stabilizers play the role of delaying the initiation stage by slowing down the generation of free radicals, prolonging the induction stage, and slowing down the degradation rate of the autocatalytic steady-state. However, the degradation of polymer modifiers in asphalt materials is inevitable and gradually worsens over time. Although the existence of stabilizers cannot completely eliminate the degradation of polymers, they play a certain role in inhibiting it.

### 3.2. Characteristics of Functional Group

**Figure 4:**
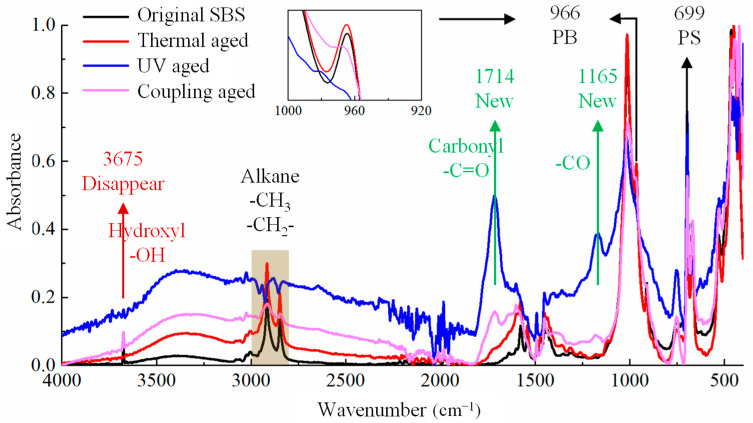
Infrared spectrum of SBS modifier under different aging conditions.

**Figure 5:**
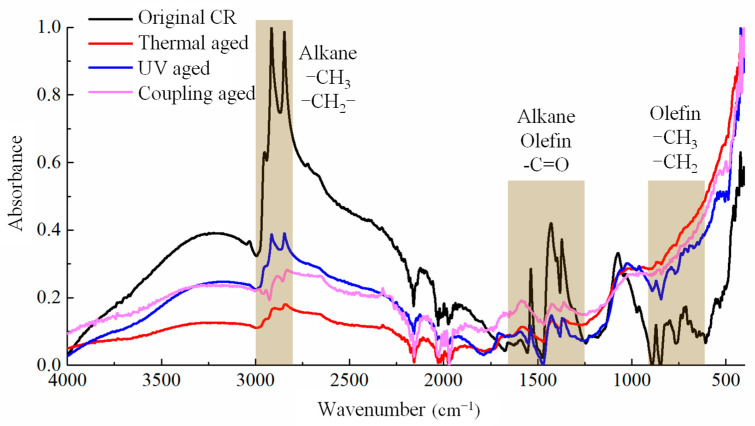
Infrared spectrum of CR modifier under different aging conditions.

The changes in the chemical composition of the two polymer modifiers are reflected in the changes in the functional group before and after aging, as shown in Figure 4 and Figure 5, respectively. Due to the significant difference in the macroscopic states before and after the aging of different modifiers, different sample preparation methods are adopted. Among them, the original SBS, UV-aged SBS, and coupled-aged SBS are still elastomers that cannot be ground into powder. Therefore, SBS particles are taken for testing, while UV-aged SBS becomes brittle and can be ground into powder for infrared testing. Similarly, the original CR and the UV-aged CR were subjected to particle testing, while thermal oxygen-aged and coupled-aged CR modifiers were ground into powder for testing.

#### 3.2.1. SBS

From Figure 4, it can be observed that the absorption peak of polybutadiene at 966 cm^−1^ and that of polystyrene near 699 cm^−1^ represent unique peaks of the SBS modifier, which can be seen clearly in the original SBS spectrogram. However, after applying different aging methods, the intensity of the peak at 966 cm^−1^ weakened significantly or even disappeared, while that at 699 cm^−1^ did not weaken significantly, indicating that the polybutadiene segment in SBS was more prone to aging and degradation under the aging effects of thermal oxygen, UV, etc. These phenomena were mainly due to the presence of unsaturated carbon–carbon double bonds in polybutadiene, which are extremely unstable and easily oxidized. The first carbon atom connected to a carbon–carbon double bond is called the *α*-Carbon. The *α*-Carbons connected to *α*-Hydrogen atoms have high activity and are the attack points for reactions.

After thermal oxygen aging, there were only slight changes in the absorption intensity of the functional groups at other positions in the SBS spectrum, the absorption peaks of 1714 cm^−1^ and 1165 cm^−1^, representing carbon–oxygen double bonds (-C=O) and carbon–oxygen single bonds (-C-O), respectively. This indicates that SBS absorbed a small amount of oxygen in the air and underwent oxidation in the thermal environment, which was related to the oxidation reaction of *α*-Hydrogen in polybutadiene.

After UV aging and coupling aging, there were significant changes in the SBS infrared spectra, not only reflected in the peak intensity changes in various functional groups, but also in the phenomenon of functional group addition or disappearance caused by chemical changes. For example, after UV aging and coupling aging, the peak intensities of the carbon–oxygen double bond (-C=O) at 1714 cm^−1^ and carbon–oxygen single bond (-C-O) at 1165 cm^−1^ were very high. SBS is a polymer based on styrene and butadiene as monomers, which do not contain O elements. The appearance of the oxygen-containing functional groups after UV aging and coupling aging meant that SBS was also affected by oxygen in the air during the UV radiation, causing the carbon–hydrogen bonds of alkanes or olefins in SBS to break, and then combine with oxygen in the air to form polymers with carbon–oxygen functional groups. The absorption peaks within this range in Figure 4 indicate the presence of -CH_3_ or -CH_2_- in alkanes. However, after UV aging and coupling aging, the peaks within this range also underwent significant irregular changes, indicating that some hydrocarbon bonds were broken, and the active groups after fracture were prone to further reaction with oxygen in air, changing the original structure of SBS.

#### 3.2.2. CR

It can be seen from Figure 5 that the positions of the absorption peaks of CR under different aging effects were basically the same, and no obvious new or missing peaks were found, which indicated that the CR powder had an anti-aging ability. CR is generally developed from waste automobile tires, which are a mixture of rubber, carbon black, and other materials, and contain an anti-aging agent. So, its anti-aging ability was stronger than that of pure SBS polymers. There was no significant change in the carbon–oxygen functional groups related to oxygen elements in the curve. However, it was also found from the spectrum that although there was no obvious new or disappeared peak, the peak intensity varied significantly in some ranges, especially in the range of 2800–3000 cm^−1^, 1250–1650 cm^−1,^ and 680–900 cm^−1^. In these ranges, the hydrocarbon bond absorption peak intensity of alkanes and olefins decreased significantly, indicating that CR experienced a degree of macromolecular carbon chain fracture degradation.

In addition, another significant difference between the infrared absorption peaks of CR- and SBS-modified asphalt was that there was no obvious peak for polybutadiene at 966 cm^−1^ and polystyrene near 699 cm^−1^. The reason is that the CR tires used in the modified asphalt industry are damaged tires of trucks or buses, which have a ratio of natural rubber (NR) to styrene-butadiene rubber (SBR) content of about 70:30, which means that only a small part of SBR contains styrene and butadiene. So, their infrared absorption peak is not strong. The main component of NR is a natural polymer compound of cis-1,4-polyisoprene with characteristic functional groups of alkanes and isoprene, whose peaks range from 1250 cm^−1^ to 1650 cm^−1^. Therefore, the absorption peak change in this range is relatively large, and its intensity significantly weakens after aging, as shown in Figure 5, indicating the occurrence of aging degradation.

### 3.3. Aging Degradation Mechanism in Polymer Modifiers

Since the main components of SBS and CR (including SBR and NR) are generated by polymerizing styrene and butadiene monomers, the aging degradation mainly occurs in the *α*-C of the PB segment. Based on the three stages of degradation in the macroscopic states of polymer modifiers and the characteristic functional groups during the aging process, the aging degradation mechanism in polymers can be inferred as follows [34,35,36].

**Figure 6:**
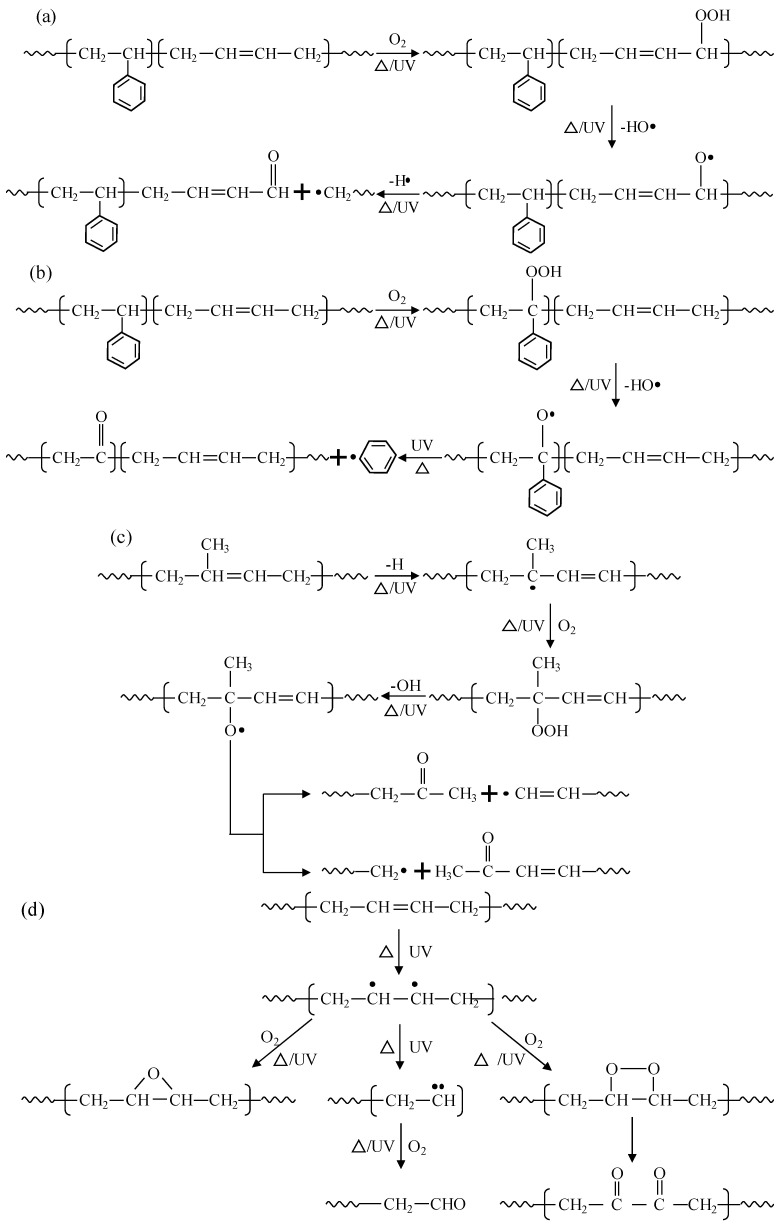
Reaction equation of degradation mechanism in polymer modifiers. (**a**) Oxidation reactions of the α-H on the C=C double bond in styrene-butadiene copolymer. (**b**) Oxidation reactions of the H on the benzene ring in styrene-butadiene copolymer. (**c**) Oxidation reactions of the C=C double bond in natural rubber. (**d**) Oxidation reaction of the C=C double bond in PB generates a dicarbonyl compound.

Under the action of heat, ultraviolet, etc., the aging degradation of a polymer starts from the *α*-H atom by absorbing oxygen to form a hydroperoxide, which is then dehydrated to form a large *π* bond-conjugated structure, and further dehydrogenates to form aldehydes or ketones, which are functional groups such as the oxygen-containing carbonyls shown in Figure 4 and Figure 5. During this process, irregular chain breaking and degradation of large molecules take place, the chemical reaction of which can be described as shown in Figure 6a. Meanwhile, the hydrogen on the carbon atoms connected to the benzene ring in the PS segment of SBS and SBR is also prone to oxidation and fracture, leading to the fracture of the benzene ring from the main chain and further ring fracture reactions, as shown in Figure 6b. However, in contrast, the *α*-H atoms in the PB segment are still more susceptible to being attacked. In addition, CR also contains a certain proportion of NR, mainly composed of cis-1,4-polyisoprene, which can undergo oxidation and degradation reactions under the action of heat, UV, etc., as shown in Figure 6c. Further, the *α*-Hydrogen atoms connected to the C=C double bond are also unstable and prone to further oxidation to form di-carbonyl compounds, as shown in Figure 6d.

## 4. Aging Mechanism of Modified Asphalt

### 4.1. Characteristics of Functional Group

#### 4.1.1. Effect of Polymer Modification on Functional Groups of Asphalts

**Figure 7:**
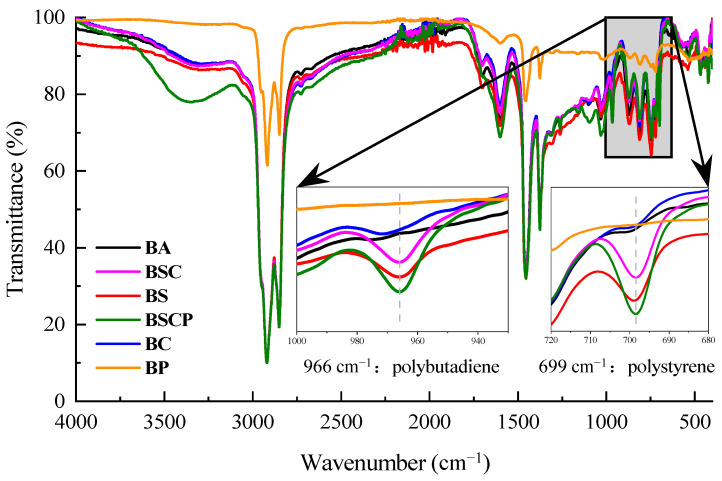
Infrared spectrum of asphaltic materials (unaged).

Figure 7 shows the infrared spectra of the five asphaltic materials in their unaged state, which reflects the influences of different polymer modifiers on the characteristic functional groups of asphalt. From the figure, it can be seen that compared to the base asphalt, the spectral curves of various modified asphalts were basically consistent, with only some positions showing the obvious characteristics of the absorption peaks of the modifiers.

Local amplification of the spectrum in the range of 1000–680 cm^−1^, as shown in Figure 7, revealed two obvious peaks after the addition of the polymer modifiers, namely the absorption peaks of polybutadiene near 966 cm^−1^ and polystyrene near 699 cm^−1^. The black curve representing the base asphalt in Figure 7 is a straight line at both positions, while the absorption peaks of different intensities appear in curves of other colors after the addition of different polymer modifiers. As for the single-polymer-modified asphalts, the intensity of the absorption peak of the SBS-modified asphalt, represented by the red curve, was significantly stronger than that of the CR-modified asphalt, represented by the blue curve, at these two points, which was consistent with the infrared spectrum of the polymer, which was modified itself, as shown in Figure 4 and Figure 5. The reason is that SBS is a compound formed solely by the polymerization of styrene and butadiene monomers. Whether it was the modifier itself or added to asphalt, the intensities of the absorption peaks at these two positions were high. However, CR is a mixture of multiple materials, with natural rubber being the main component and SBR being relatively small. Therefore, the strengths of the absorption peaks of polystyrene and polybutadiene in CR and its modified asphalt were relatively weak. The absorption peaks of polystyrene and polybutadiene in both the SBS/CR composite-modified asphalt (BSC) and the plasticized SBS/CR composite-modified asphalt (BSCP) were strong due to the presence of both polymer modifiers.

For comparison, PPA-modified asphalt (BP, prepared from base asphalt with 1.5% polyphosphoric acid) was introduced as a reference to represent non-polymer modification systems. Unlike SBS/CR-based modifications that involve complex polymer chain segments susceptible to degradation, PPA modification is characterized by small-molecule functional groups (primarily P=O) without any elastomeric polymer structure. As shown in Figure 7, the PPA-modified asphalt exhibited no discernible absorption peaks in the hydroxyl absorption peak (3700–3200 cm^−1^), at the polybutadiene absorption peak (966 cm^−1^), or at the polystyrene absorption peak (699 cm^−1^). This absence of characteristic polymer peaks methodologically validated its role as a control group, highlighting the distinct structural complexity of SBS/CR-modified asphalt. As PPA contains no organic polymer segments with C=C bonds or α-H susceptible to oxidative degradation, it was excluded from subsequent aging experiments that specifically targeted the degradation mechanisms of elastomer-modified asphalts.

The enhanced effects of the absorption peaks of polystyrene and polybutadiene modified with SBS and CR can also be obtained from the quantitative calculation of their spectra, which were obtained using the OMNIC V8.2 software for two locations of the five asphaltic materials under consideration, as shown in Table 3. The intensity of an absorption peak of a functional group refers to the corrected area enclosed by the absorbance curve of the infrared spectrogram and baseline within a specific range. The calculated intensity range of the polystyrene peak was 690–708 cm^−1^, and that of the polybutadiene peak was 950–983 cm^−1^. Similarly to the directly observed results, the strength of the matrix asphalt was the lowest at the two absorption peaks of polystyrene and polybutadiene, only 0.033 and 0.057, followed by the CR-modified asphalt, with slightly higher strength at both peaks than the matrix asphalt. The two peak strengths of the SBS-modified asphalt were significantly stronger than those of the first two types of asphalt. In addition, the absorption peak strengths of the composite-modified asphalts increased compared with that of the SBS-modified asphalt, while those of the plasticized SBS/CR composite-modified asphalt was the largest, both of which were above 0.8, which could be attributed to the pre-swelling of the plasticizer to CR, and the reactive groups in the plasticizer reacted easily with the isoprene group of natural rubber in CR, resulting in a reduction in the natural rubber content in CR. The content of SBR, polymerized from styrene and butadiene, was relatively increased. The addition of plasticizer allowed the CR to more fully swell, dissolve, and disperse in the asphalt. Therefore, strong absorption peaks of polystyrene and polybutadiene could be detected in the plasticized SBS/CR composite-modified asphalt.

#### 4.1.2. Effect of Aging on Functional Groups of Asphalts

**Figure 8:**
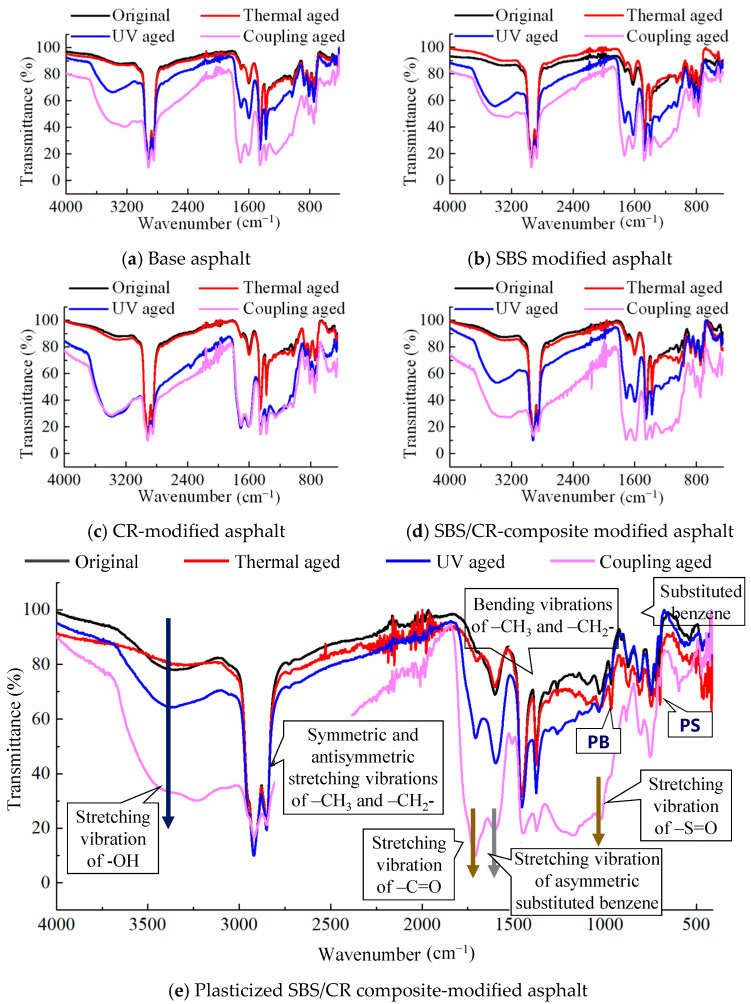
Infrared spectra of five asphaltic materials under different aging conditions.

The results of the infrared spectrum test of the five asphaltic materials under different aging effects are shown in Figure 8. Intuitively, the infrared spectrum of the asphaltic materials after short-term aging almost coincided with the original asphalt, with only a few absorption peaks of oxygen-containing functional groups showing slight changes. However, the infrared spectrogram of asphalt changed significantly after UV aging and coupling aging. Due to the radiation of high energy ultraviolet rays, most chemical bonds in the asphaltic materials, such as the C-H bond, C-C bond and C=C double bond, were easy to break down to produce reactive free radicals, which could react with asphalt or other components in the modifier, and change the strengths of the absorption peaks of most functional groups in the plasticized SBS/CR composite-modified asphalt.

Due to the consistent changes in the infrared spectra of the five asphaltic materials under different aging effects, the most complex plasticized SBS/CR composite-modified asphalt was taken as an example for specific analysis. As shown in Figure 8e, the infrared spectrum of the plasticized SBS/CR composite-modified asphalt was annotated with some functional groups corresponding to the characteristic absorption peaks. Local amplification of the spectrum revealed that although the absorption peaks appeared to have undergone significant changes on the surface after UV aging and coupling aging, in reality, most of them only enhanced or weakened the peak intensity or transfer, with the appearance of few new functional groups. The summary of these changes is reported in Table 4. Compared to the original plasticized SBS/CR composite-modified asphalt, there were new peaks near 1710 cm^−1^ under different aging methods, which were typical absorption peaks of carbonyl C=O in the oxidative aging group. The sulfoxide S=O group at the 1030 cm^−1^ position, another characteristic peak of the oxidation aging, was not new. It also appeared in the original plasticized SBS/CR composite-modified asphalt. But its peak strength increased after aging, indicating that the sulfoxide group appeared earlier in the preparation process of the plasticized SBS/CR composite-modified asphalt due to the reaction between the asphalt and oxygen in the air. Additionally, carbonyl growth is modest in thermal aging but accelerates markedly under UV exposure.

In addition, there were two more characteristic peaks, namely the absorption peaks of the polymer modifiers, such as SBS and CR, at positions 966 cm^−1^ and 699 cm^−1^, respectively. After aging, the peak at position 966 cm^−1^ significantly weakened or even tended to disappear, while that at position 699 cm^−1^ also experienced a certain degree of intensity attenuation. This result was consistent with the infrared spectra of SBS and CR after individual aging, indicating that the implementation of thermal, UV, and other aging effects produced a certain degree of oxidative aging and degradation on the asphalt and polymer modifiers.

### 4.2. Aging Mechanism of Asphaltic Materials

The first consideration of aging for asphaltic materials is their oxidation, as compared to their physical hardness. The impact of oxidation, which may affect the chemical and physical properties of asphalt, is irreversible. The influence of aging on the light components of asphalt is more important than natural evaporation. From a chemical perspective, the asphalt aging is a process of component oxidation.

**Figure 9:**
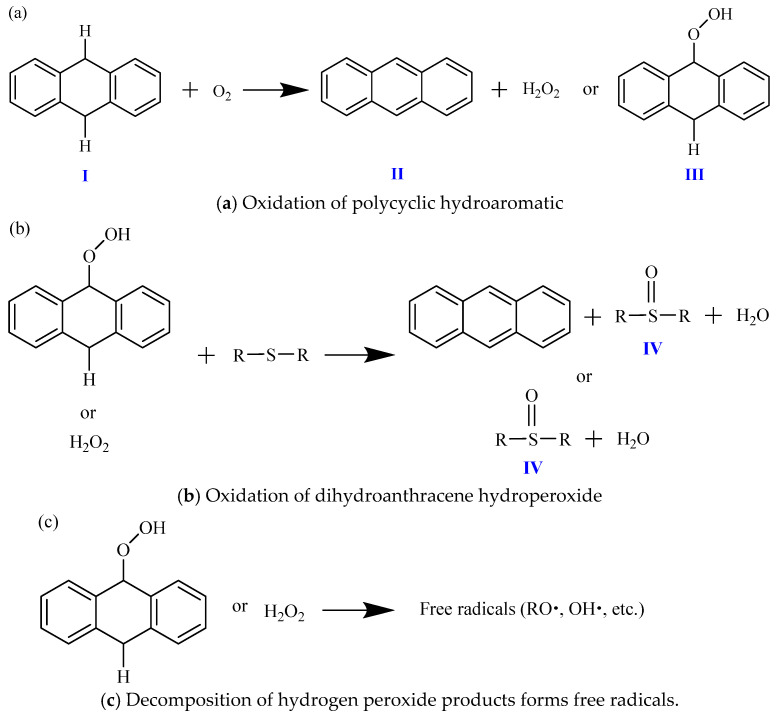
Reaction progress of oxidation of polycyclic perhydroaromatics in asphalt (illustrated for dihydroanthracene).

Taking dihydroanthracene as an example, the oxidation reaction process in the early stage of aging is described as follows. Firstly, after the oxidation of polycyclic hydroaromatic hydrocarbons (I), hydrogen peroxide (II) or hydroaromatic hydrogen peroxide (III) can be produced, as shown in Figure 9a. Among them, dihydroanthracene hydroperoxide (III) accounts for about 90% of the oxidation reaction, and only about 10% of the reaction generates hydrogen peroxide (II). Then, these two products can react with active sulfides (sulfur ether, aliphatic sulfide, thiophene, or aromatic sulfide) in asphalt to generate the sulfoxide group (IV) and aromatize the hydrogen aromatics. Therefore, the oxidation of hydrogen aromatics in asphalt increases the aromaticity of asphalt, as shown in Figure 9b, which further proves that asphalt is more aromatized after aging. In addition, during the sudden increase in the oxidation reaction, some hydrogen peroxide products may undergo decomposition to form free radicals, as shown in Figure 9c. These free radicals may then initiate and increase the chain oxidation reaction of benzyl carbon to generate carbonyl functional groups (such as ketones). Due to the temperature sensitivity of hydrogen peroxide decomposition, they may decompose into free radicals faster at higher temperatures. It helps asphalt to generate carbonyl groups faster during the sudden increase in the oxidation reaction, resulting in a faster oxidation rate and a higher degree of aging of asphalt at high temperatures [37,38,39,40].

**Figure 10:**
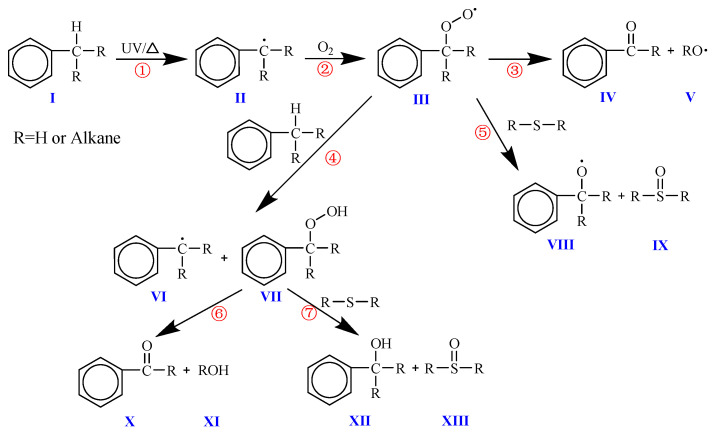
Carbonyl formation during oxidation of asphalt benzylic carbon.

Figure 10 shows the possible oxidation reaction process of the benzyl compounds contained in asphalt. The oxidation of benzyl carbon (I) first occurs when the benzyl hydrogen connected to it is excited into a free state under thermal or ultraviolet action (①) to form benzyl (II), and then the benzyl reacts with oxygen (②) to form peroxybenzyl (III). The peroxy radical is the most stable radical formed in a typical hydrocarbon chain transfer reaction. It may be the main precursor structure of ketone (carbonyl), which may further react (③) to produce ketone containing carbonyl (IV) and other free radicals (V). In addition, during the sudden increase in the benzyl carbon oxidation (④, VI), the most likely pathway for the formation of sulfoxides in asphalt is actually the reaction between the benzyl hydrogen peroxide (VII) and asphalt alkyl or alkyl aryl sulfides (⑤, ⑦). During the reaction with sulfides, oxygen is transferred, and sulfides are promoted to sulfoxides (IX, XIII) and benzoyloxy (VIII, XII) through synchronous hydrogen atom exchange. In addition, the decomposition of benzyl hydrogen peroxide (VII) (⑥) is another important reaction pathway for the formation of carbonyl ketones (X) and hydroxide (XI) [41].

Therefore, the oxidation reaction of the asphalt in this research is a dual sequential oxidation process. In the early stage of the reaction, especially under low temperature, hypoxia, and diffusion-controlled conditions, a large number of sulfoxide groups are formed during the oxidation aging. That is, the asphalt oxidation begins with the reaction of highly active hydrocarbons. Therefore, after long-term storage at room temperature, asphaltic materials undergo certain aging phenomena even without heating and UV aging, with the main product being sulfoxide groups. During the initial stage of the reaction, no carbonyl group is generated, as shown in Figure 9b. In the later stage of the oxidative aging reaction, as the strength of the active free radicals increases, the oxidation reaction of asphalt benzyl carbon also begins to significantly enhance, ultimately generating carbonyl groups, as shown in the reaction process in Figure 10. Therefore, asphalt reacts to generate sulfoxide and carbonyl groups under long-term storage, short-term aging, and long-term aging conditions. However, the aging products and yields vary at different stages. When oxygen-containing functional groups are formed in asphalt and their contents increase, the components of asphalt transition from non-polar to polar substances.

## 5. Conclusions

This paper applied aging effects to different modifiers and their modified asphalt, and revealed the aging mechanism of composite modified asphalt through FTIR-ATR from the perspectives of aging oxidation and chemical degradation. The main conclusions are as follows:

(1) SBS and other aromatic polymer modifiers are highly sensitive to high-temperature thermal effects and UV radiation, and they are prone to yellowing and degradation. *α*-Hydrogen atoms, which are highly active in modifiers, are prone to oxygen absorption and aging, leading to chain breakage under high-temperature and UV conditions, degradation, and the generation of some active groups and oxygen-containing compounds such as carbonyls.
(2) The aging reaction of the asphalt in this research is a dual sequential oxidation process. In the early stage of the reaction, especially under low temperature, oxygen deficiency, and diffusion-controlled conditions, a large amount of sulfoxide groups are formed. That is, the oxidation of asphalt begins with the reaction of highly active hydrocarbons. Therefore, after long-term storage at room temperature, asphalt undergoes aging phenomena even without heating and UV aging, with the main product being sulfoxide groups. In the later stage of the oxidative aging reaction, as the strength of active free radicals increases, the oxidation reaction of asphalt benzyl carbon also begins to enhance significantly, ultimately generating carbonyl groups.
(3) Asphalt inevitably undergoes aging reactions to generate sulfoxide and carbonyl groups under long-term storage, short-term heating, and long-term service conditions. However, the aging products and yields vary at different stages. Therefore, the aging behavior of polymer-modified asphalt cannot be completely solved, and further physical and chemical modifications are needed to delay the aging reaction, so as to improve the anti-aging performance and service life of modified asphalt.

This paper only analyzed the modifying effects of SBS and CR modifiers on asphalt and the aging mechanism of modified asphalt in a multi-source environment through FTIR. Subsequent research will employ more testing and analysis techniques, such as NMR or GC-MS, to comprehensively evaluate the aging behavior of modified asphalt.

## Figures and Tables

**Table 1:** Basic properties of raw materials.

Material	Technical Performances		Technical Requirement
Base asphalt	Density (15 °C, g/cm^3^)	1.015	—
	Penetration (25 °C, 0.1 mm)	84.1	80~100
	Softening point (TR&B, °C)	45.4	≥43
	Ductility (5 cm/min 10 °C, cm)	>100	≥100
SBS	Block ratio (S/B)	30/70	—
	Average molecular weight	95,000	—
	Ash content (%)	0.09	≤0.2
	Tensile strength (MPa)	23.6	≥15
	Elongation (%)	752	≥700
CR	Relative density	1.13	1.10~1.30
	Natural rubber content (%)	31	≥25
	Ash content (%)	5.6	≤8
	Carbon black content (%)	29.7	≥28
	Rubber hydrocarbon content (%)	57.2	≥42
Plasticizer	Appearance	Colorless to yellow oily liquid	—
	Purity (%)	99.5	—
	Chemical name	Furfural	
	Chemical formula	C_5_H_4_O_2_	—
	Relative density	1.16	—
Stabilizer	Appearance	Gray solid fine powder	—
	Active ingredient content (%)	≥95	—
	Apparent density (g/cm^3^)	0.6~0.8	—
	Melting point (°C)	≥110	—

**Table 2:** Composition of raw materials used in different modified asphalts.

Abbreviation	Base Asphalt	SBS	CR	Plasticizer	Stabilizer	Notes
BA	100%	—	—	—	—	Base Asphalt
BS	100%	6%	—	—	0.36%	Base asphalt + SBS
BC	100%	—	25%	—	0.36%	Base asphalt + CR
BSC	100%	6%	25%	—	0.36%	Base asphalt + SBS + CR
BSCP	100%	6%	25%	15%	0.36%	Base asphalt + SBS + CR + Plasticizer

**Table 3:** Intensities of infrared absorption peaks of polystyrene and polybutadiene in unaged asphaltic materials.

Asphalt Type	Polystyrene Peak Strength (690–708 cm^−1^)	Polybutadiene Peak Strength (950–983 cm^−1^)
Base asphalt (BA)	0.033	0.057
SBS-modified asphalt (BS)	0.435	0.452
CR-modified asphalt (BC)	0.052	0.209
SBS/CR composite-modified asphalt (BSC)	0.481	0.597
Plasticized SBS/CR composite-modified asphalt (BSCP)	0.807	0.890

**Table 4:** Functional groups of sealant and their corresponding peak positions.

Peak Position (cm^−1^)	Characteristic Functional Groups	Trend
3327	Broad band associated with O–H/N–H stretching	Significantly enhanced after aging. Changes in oxygen-containing functional groups were clearly reflected in both aging of the modifier alone and aging of the modified asphalt, while polysubstituted benzene had only higher strength in modified asphalt
1712	Stretching vibration of carbonyl (-C=O), ketones or aldehydes	
1030	Stretching vibration of sulfoxide (-S=O)	
1600	Stretching vibration of asymmetrically substituted benzene	
873	Stretching vibration of benzene ring, pentasubstituted benzene	
812	Stretching vibration of benzene ring, disubstituted and trisubstituted benzene	
966	Polybutadiene (-C=C-)	Weakening or disappearance after aging. It was clearly reflected in both aging of the modifier alone and aging of the modified asphalt
910	Olefin monosubstitution	
721	Resonance of -(CH_2_)_n_-, *n* ≥ 4	
699	Polystyrene, monosubstituted benzene	

## Data Availability

The original contributions presented in this study are included in the article. Further inquiries can be directed to the corresponding authors.
